# Emotional Processing Impairments in Apathetic Patients with Parkinson's Disease: An ERP Study in Early Time Windows

**DOI:** 10.1155/2019/1309245

**Published:** 2019-04-15

**Authors:** Wei Wei, Jianghai Ruan, Xiaodong Duan, Hua Luo

**Affiliations:** ^1^Department of Neurology, The Affiliated Hospital of Southwest Medical University, Luzhou 646000, Sichuan, China; ^2^Department of Rehabilitation Medicine, The Affiliated Hospital of Southwest Medical University, Luzhou 646000, Sichuan, China

## Abstract

We investigated emotional processing in apathetic patients with Parkinson's disease (PD) by observing components of event-related potentials (ERPs) in early time windows. Forty PD patients and 21 healthy controls (HCs) were enrolled. The Starkstein Apathy Scale (SAS) was used to divide the PD patients into apathetic and nonapathetic groups. Cognitive function was evaluated by the forward and backward Digit Span tests, Trail Making Test (TMT), and Word Fluency Test. The participants were required to recognize positive, neutral, and negative emotional faces and engage in an emotion categorization task while EEG was recorded. The time to completion for the TMT (Part A and Part B) from highest to lowest was in the order of apathetic group > nonapathetic group > HC group. Compared with the nonapathetic and HC groups, in the apathetic group, P100 amplitudes were smaller for positive expressions in the right hemisphere and latencies were longer for positive expressions in the left hemisphere, while latencies were longer for neutral expressions bilaterally. Compared with the nonapathetic group, in the apathetic group, N170 amplitudes were attenuated and latencies were delayed for neutral and negative expressions in the right hemisphere. A trend towards larger N170 amplitudes in the right hemisphere than in the left was observed in the nonapathetic and HC groups, but this difference was not significant in the apathetic group. In the apathetic group, bilateral P100 amplitudes elicited by negative expressions were negatively correlated with SAS scores, and SAS scores were positively correlated with Part B of the TMT. N170 amplitudes elicited by negative expressions in the right hemisphere were negatively correlated with SAS in the apathetic group and with Part B of TMT in both PD groups. Our findings suggested that emotional processing was impaired in apathetic PD patients and that the right hemisphere was more sensitive to reflecting this impairment in the early time windows of ERPs.

## 1. Introduction

Apathy is one of the most important nonmotor symptoms in Parkinson's disease (PD), and it is associated with the neurocognitive impairment caused by PD [1]. Pure apathy is considered apathy without comorbid depression and dementia [2] and has been identified as a reduction in goal-directed behaviour that shows a lack of motivation, feeling, interest, and emotion [3]. Pure apathy is associated with impaired daily function and quality of life and increased stress for caregivers. Dopaminergic depletion, especially in the nigrostriatal system, was noted to play a key role in the development of apathy [1]. Apathy is caused by dopamine depletion in the ventral striatum, which is important for the modulation of emotional behaviours by the frontal cortex [4]. In addition to the frontal cortex, apathy has been associated with the precuneus [5] and the right medial temporal lobe [6] in PD patients. The precuneus has been associated with a variety of psychological dysfunctions, such as depression [7], obsessive-compulsive disorder [8], and schizophrenia [9]. The precuneus was modulated by unpleasant stimuli in normal subjects assessed by magnetoencephalography [10] and was associated with the allocation of attention to emotional faces [11]. The right medial temporal lobe is responsible for implicit processing during object recognition [12] and is correlated with cognitive impairment [13, 14].

Event-related potentials (ERPs) have been useful for investigating emotional processing by affective facial stimuli because of their excellent temporal resolution. P100 reflects basic visual processing, appears at approximately 70–130 ms in normal subjects, and is mostly detected at occipital sites. N170, which is a large negative deflection elicited by face perception, appears at approximately 130–200 ms post stimulus and reflects the structural encoding stage. The N170, which is considered to be sensitive to affective faces [15–17], can be used as an objective measure of face perception [18]. The latency of the response following emotional face presentation indicates the timing of emotional processing, and the amplitude indicates the intensity. Different components of the ERP were associated with apathy [19, 20], but studies of ERP components in early time windows modulated by emotional processing in apathetic subjects are limited. According to previous studies, there were emotional processing deficits in apathy [21, 22]. Both P100 and N170 reflect emotional intensity at an early stage of visual processing [23], and these potentials are modulated by emotional facial expressions [24]. Therefore, we hypothesized that the early ERP components that reflect emotional processing may be influenced by apathy in PD. In this study, we aimed to investigate the relationships between emotional processing and apathy using electrophysiological and neuropsychological measures.

## 2. Materials and Methods

### 2.1. Participants

This study protocol was reviewed and approved by the Ethics Committee of the Affiliated Hospital of Southwest Medical University. Each of the participants provided informed consent (in the Chinese language) before participating in this study. In most cases, the participants provided their written informed consent.

A total of 40 PD outpatients and inpatients meeting the UK Brain Bank criteria for idiopathic Parkinson's disease [25], and 21 healthy older adults were selected from the Affiliated Hospital of Southwest University. The motor scores from the Unified Parkinson's Disease Rating Scale (UPDRS) [26] and Hoehn–Yahr classification [27] were obtained by experienced neurologists. Depression was assessed by the Beck Depression Inventory (BDI-II) [28]. All PD patients were at an early or middle stage of PD according to the Hoehn–Yahr classification and were confirmed to have normal sight. The PD patients were grouped into an apathetic group and a nonapathetic group based on the Starkstein Apathy Scale (SAS) (cutoff score for apathy was ≥14) [29]. There were 15 patients in the apathetic group and 25 patients in the nonapathetic group. All patients were undergoing anti-Parkinsonian treatment. There were 9 apathetic patients and 14 nonapathetic patients taking L-DOPA, 3 apathetic patients and 7 nonapathetic patients taking dopamine agonists, and 3 apathetic patients and 4 nonapathetic patients taking MAO inhibitors. The current daily L-DOPA doses were calculated using MacDonald's formula [30]. The exclusion criteria were as follows: (1) advanced PD; (2) depression (Beck Depression Inventory II (BDI-II) score > 14 (mild depression)); (3) cognitive impairment (Montreal Cognitive Assessment (MoCA) < 26); (4) focal abnormalities in neuroimaging; (5) history of other neurological conditions or surgery; (6) unstable response to dopaminergic medications. The HCs were recruited from the health examination department, and there was no significant difference in age, sex ratio, or education between the groups.

### 2.2. Neuropsychological Tests

All the subjects were assessed by a series of neuropsychological tests. To evaluate the subjects' working memory, the forward and backward Digit Span tests [31] were used. Executive function was assessed by the Trail-Making Test (TMT) [32], including Part A and Part B. The Word Fluency Test [33] required participants to provide as many fruit/vegetable and animal words as possible within 1 minute.

### 2.3. Stimuli and Task

**Figure 1:**
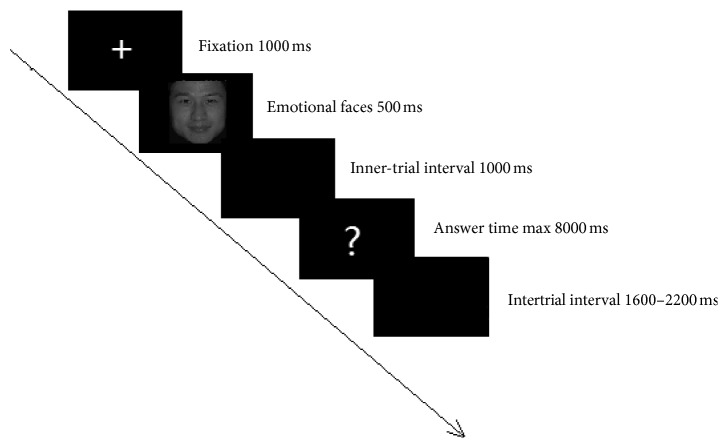
Schematic representation of a typical trial in the experiment.

Stimuli consisted of a standardized set of emotional facial expressions selected from the Chinese Facial Affective Picture System database [34], which contains positive, neutral, and negative (fear, anger, sadness, and disgust; 8 pictures for each expression) expressions. There were no significant differences in the intensity among the positive (5.84 ± 0.30), neutral (5.94 ± 0.12), and negative expressions (5.91 ± 0.24) (*F* = 1.571, *p*=0.213). There were 32 black and white pictures for each expression, and there were no significant differences in emotional intensity. Each trial began with a white fixation cross presented for 1 second. Then, each picture was presented for 500 ms in random order in the centre of the black background subtending a visual angle of approximately 11° to 15°, and instructions appeared after 1000 ms that required the subjects to identify the expression by pressing the corresponding button as quickly as possible. The instructions would not disappear unless the subjects reacted or a maximum of 8000 ms had passed. After the presentation of a blank screen for a random duration between 1600 and 2200 ms, the next trial began (Figure 1). EEG was recorded during the task. Wolwer et al. [35] applied this method in a previous study.

### 2.4. ERP Recording and Processing

EEGs were recorded from 32 electrodes according to the 10/20 system using BrainAmp MR (Brain Products, Germany) and digitized at 500 Hz. Electrode impedances were kept under 10 kΩ, and the recording was referenced to the common average.

The horizontal and ocular artefacts were corrected, and excessive EEG artefacts were identified by visual inspection and excluded. The trials were trimmed to 200 ms before through 600 ms after presentation of the stimulus, and pre-stimulus baseline was corrected to 200 ms. The amplifier bandpass frequency was 0.1–70 Hz with a sampling rate at 250 Hz, and trials with amplitudes exceeding ±75 were rejected.

P100 (70∼130 ms) and N170 (150∼200 ms) components were averaged at occipital (O1 and O2) and lateral parietal (P7 and P8) sites.

### 2.5. Statistical Analysis

The statistical analysis was performed using SPSS software (Version 24.0, SPSS Inc., Chicago, IL, USA). The differences in demographic characteristics and neuropsychological tests were assessed using the nonparametric Kruskal–Wallis test and chi-square test. The behavioural results were analysed with a repeated measures analysis of variance (ANOVA) with the factors Group (apathetic PD, nonapathetic PD, and controls) × Emotion (positive, neutral, and negative). The latency and amplitude of P100 and N170 were analysed by repeated measures ANOVA with Group × Emotion × Hemisphere (left and right) as within-subjects factors. ANOVA *p* values were calculated with the Greenhouse–Geisser correction in case of violation of sphericity.

## 3. Results and Discussion

### 3.1. Demographic Data and Clinical and Test Scores

Among all the subjects, 40 patients and 21 controls completed all the examinations and tests. There were 15 apathetic and 25 nonapathetic patients in the case group, and there were no significant differences in Hoehn–Yahr classification, UPDRS scores, L-DOPA daily dose, and disease duration. There were no significant differences among the three groups with respect to age, sex, education, and MoCA, BDI-II, SAS, Digit Span, or Word Fluency scores. The TMT showed that the apathetic group performed significantly worse than the nonapathetic and HC groups in both Part A and Part B (Table 1).

### 3.2. Behavioural Results

A Group × Emotion ANOVA was performed on accuracy (ACC). There was a significant Group × Emotion interaction (*F*4,116 = 10.411, *p* < 0.001, *η*_*p*_^2^ = 0.264). Since accuracy was not normally distributed, we used nonparametric tests to analyse the differences among the groups. There was no difference in the three groups for ACC in positive expression trials, while it was lower in the PD groups than that in the HC group in neutral and negative expression trials. ACC was lower in the apathetic group than that in the nonapathetic group in negative expression trials.

A Group × Emotion ANOVA was performed on reaction time (RT). A significant interaction of emotion was observed (*F*2,116 = 17.574, *p* < 0.001, *η*_*p*_^2^ = 0.233). The difference among the three groups was analysed by post hoc ANOVA. Similar to ACC, there was no difference in the three groups for RT in the positive expression trials, while it was lower in the PD groups in the neutral and negative expression trials, and there was no significant difference between the apathetic and nonapathetic groups (Table 2).

### 3.3. P100

For the amplitude, there was a significant effect of emotion (*F*2,116 = 5.712, *p*=0.004, *η*_*p*_^2^ = 0.090). Additionally, a significant effect of Hemisphere × Group was observed (*F*2,58 = 13.342, *p* < 0.001, *η*_*p*_^2^ = 0.315). The amplitude in the PD groups for all expressions was lower than that in the HC group in the left hemisphere, while there was no significant difference between the apathetic and nonapathetic PD groups. In the right hemisphere, the amplitude was significantly lower in the apathetic group than in the nonapathetic (*p*=0.01) and HC (*p*=0.048) groups for positive expressions, and there was no significant difference in the three groups for neutral and negative expressions.

**Figure 2:**
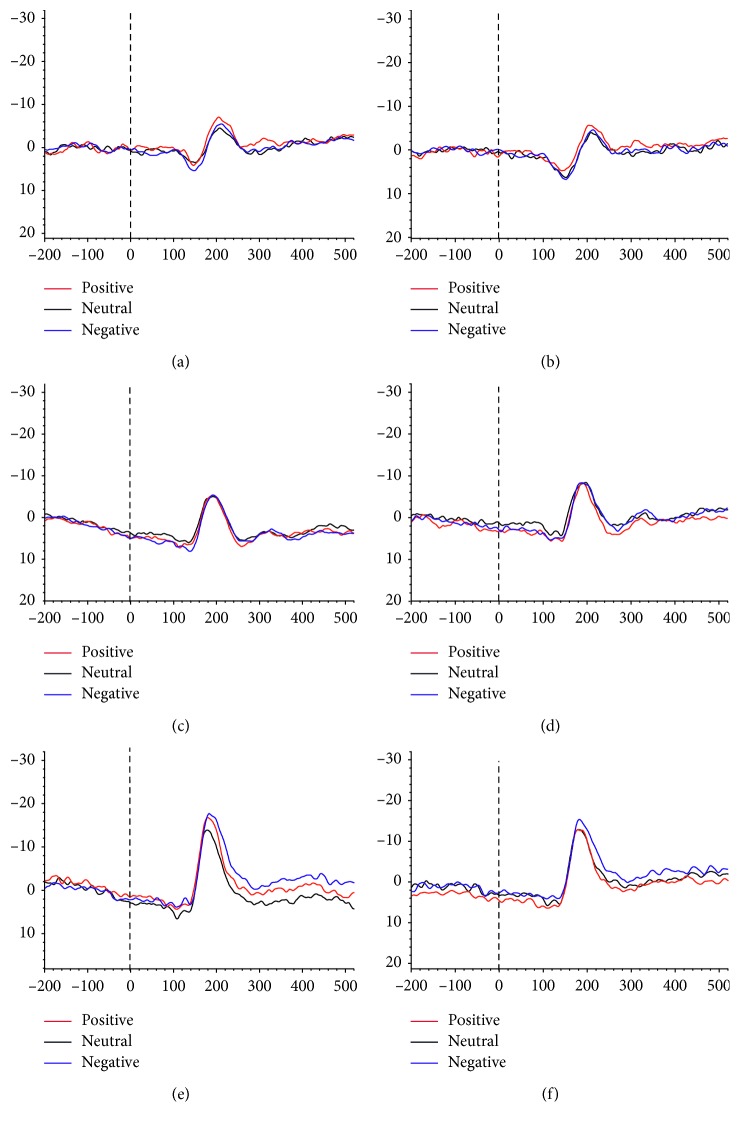
Grand-averaged event-related potential (ERP) waveforms elicited at left and right occipital electrodes (O1 and O2). (a) ERP waveforms of O1 in the apathetic group. (b) ERP waveforms of O2 in the apathetic group. (c) ERP waveforms of O1 in the nonapathetic group. (d) ERP waveforms of O2 in the nonapathetic group. (e) ERP waveforms of O1 in the HC group. (f) ERP waveforms of O2 in the HC group.

The latency of the apathetic group was longer than that of the nonapathetic and HC groups for positive (*p*=0.003) and neutral (*p* < 0.001) expressions in the left hemisphere and for neutral expressions (*p* < 0.001) in the right hemisphere. The latency was longer in both PD groups than in the HC group for negative (*p*=0.023) expressions in the left hemisphere and for positive (*p* < 0.001) and negative (*p*=0.001) expressions in the right hemisphere (Figure 2, Table 3).

### 3.4. N170

For the amplitude, a significant main effect of the hemisphere was observed. The amplitude was larger for the right hemisphere (P8) than that for the left hemisphere (P7) (*F*1,58 = 25.651, *p* < 0.001, *η*_*p*_^2^ = 0.307). Further analysis indicated that there was a significant main effect of the hemisphere in the nonapathetic (*F*1,24 = 28.667, *p* < 0.001) and HC (*F*1,20 = 13.506, *p*=0.002) groups, but there was no significant effect of the hemisphere in the apathetic group. N170 amplitudes were significantly smaller in the PD groups than those in the control group. The amplitude was attenuated in the apathetic group compared with the nonapathetic group in the right hemisphere for neutral (*p*=0.043) and negative expressions (*p*=0.016). The N170 amplitude in the right hemisphere compared with the left hemisphere was significantly higher for positive expressions in the nonapathetic group (*p*=0.038) and for neutral expressions in the nonapathetic (*p*=0.026) and HC (*p*=0.002) groups. There was a trend towards a significant difference in positive expressions in the HC group (*p*=0.074) and negative expressions in the nonapathetic (*p*=0.051) and HC (*p*=0.052) groups, while there was no difference between the right and left hemispheres for all expressions in the apathetic group (all *p* > 0.05).

**Figure 3:**
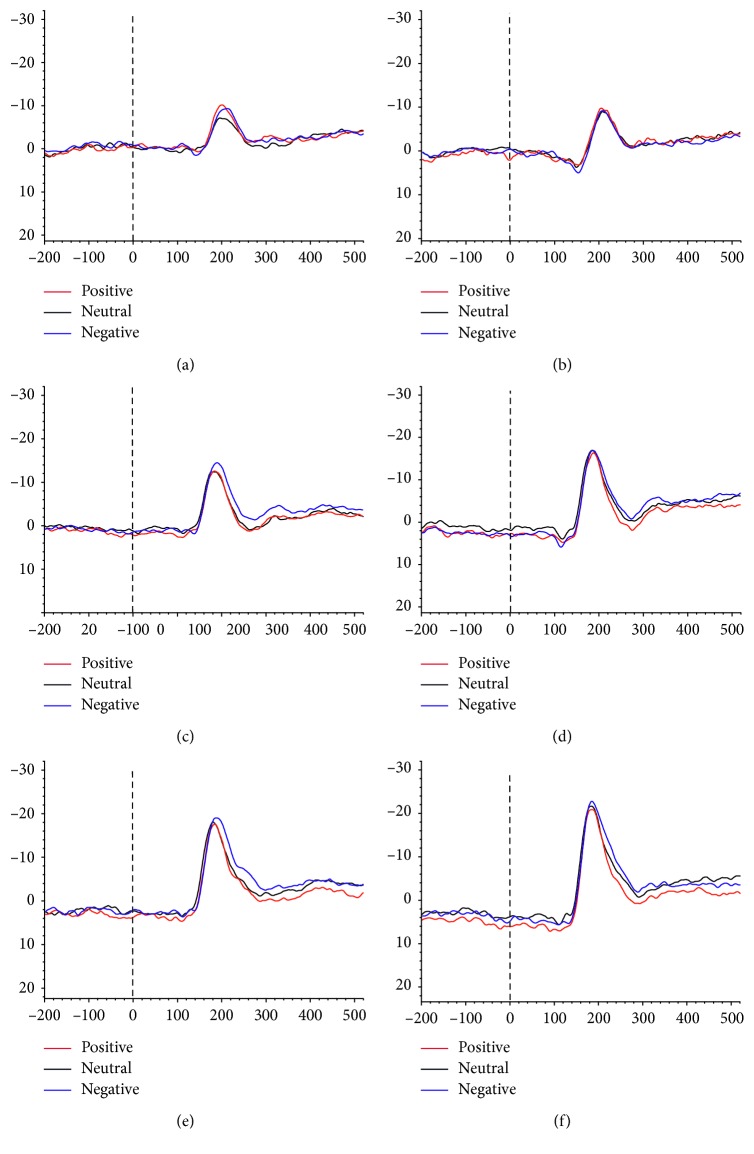
Grand-averaged ERP waveforms elicited at left and right temporo-occipital electrodes (P7 and P8). (a) ERP waveforms of P7 in the apathetic group. (b) ERP waveforms of P8 in the apathetic group. (c) ERP waveforms of P7 in the nonapathetic group. (d) ERP waveforms of P8 in the nonapathetic group. (e) ERP waveforms of P7 in the HC group. (f) ERP waveforms of P8 in the HC group.

For latency, a significant interaction of Group × Hemisphere × Emotion was observed (*F*4,116 = 3.208, *p*=0.015, *η*_*p*_^2^ = 0.10). According to the post hoc Tukey's test, there was a longer latency in the apathetic PD group than in the nonapathetic PD group in the right hemisphere for neutral and negative expressions. There was no significant difference between the nonapathetic PD and HC groups for latency (Figure 3, Table 3).

### 3.5. Correlations between Apathy, Neuropsychological Tests, and ERPs

The P100 amplitudes for negative expressions in the left hemisphere (*r* = −0.622, *p*=0.013) and right hemisphere (*r* = −0.518, *p*=0.048) were negatively correlated with the SAS score in the apathetic PD group. There was no correlation between P100 amplitude or latency and SAS scores in other groups.

The N170 amplitude for negative expressions in the right hemisphere negatively correlated with the SAS score (*r* = −0.577, *p*=0.024 in the apathetic group; *r* = −0.426, *p*=0.034 in the nonapathetic group) and Part B of the TMT (*r* = −0.692, *p*=0.004 in the apathetic group; *r* = −0.446, *p*=0.025 in the nonapathetic group) in the PD groups, while no significant correlation was found in the HC group. There was no significant correlation between N170 latency and apathy scores and neuropsychological tests in all groups.

The SAS score was positively correlated with Part B of the TMT in the apathetic group (*r* = 0.525, *p*=0.044). No significant correlation was found in other neuropsychological tests.

## 4. Discussion

### 4.1. Executive Function and Behavioural Performance

In this study, we aimed to investigate emotional processing in apathetic PD patients using an analysis by the early time windows of ERP combined with neuropsychological tests. Deficits in executive function reflect frontostriatal dysfunction [36] and can be reversed by dopaminergic medications [37]. As apathetic PD patients show decreased functional connectivity in the frontostriatal cortex [38], there may be an association between executive function and apathy. Since executive function is estimated by the TMT, the poorer scores on the TMT of apathy patients are consistent with this interpretation. The association is also implied in the positive correlation of SAS and Part B of the TMT. Similar to a previous study, we found that PD patients had problems categorizing emotional facial expressions evidenced by the longer RT and lower accuracy for negative and neutral faces [39]. The most likely reason for similar RTs and accuracy for the identification of positive faces in all subjects was that happy faces are easier to categorize than negative and neutral faces [40]. Similarly, Narme et al. [41] showed that PD patients had impaired emotion recognition only for angry and fearful faces. There were no significant differences in RT for the identification of all expressions between the apathetic and nonapathetic patients, indicating the low sensitivity of RT. The lowest ACC for negative expressions of apathetic patients showed the worst emotional processing. Frontal and temporal areas have been implicated in apathy in PD patients [42], and these areas play a key role in emotional processing [43], which may also explain the poorer emotional recognition in apathetic PD patients.

### 4.2. Event-Related Potentials

The amplitude of P100 may be associated with emotion [44]. This study showed a smaller P100 amplitude for all expressions in the two PD groups than in the HC group in the left hemisphere, suggesting a possible bluntness to visual processing in the case of PD. Although there was no significant difference in the behavioural results for positive expressions among the three groups, the amplitude in the right hemisphere for positive expressions was particularly smaller in apathetic patients than that in nonapathetic patients and HCs, implying a shift away from automatically processing positive expressions. The longest latency of apathetic patients for positive expressions in the left hemisphere and neutral expressions in the bilateral hemisphere also showed the worst P100 results, demonstrating the lower sensitivity to rapid emotional processing in these patients. The primary coding of visual configuration is mediated by the occipital lobe [45], and apathy is correlated with the hypoperfusion of the occipital lobe according to previous studies [46]. In this study, the association of P100 amplitude and SAS scores in apathetic patients indicates that early emotional processing and apathy may share a common neural mechanism.

The N170 amplitude in PD patients can be increased by levodopa treatment, indicating an association of dopaminergic depletion and the early stages of emotional processing [47] and may support the poorer N170 indexes in apathetic patients in this study. Notably, the nonapathetic PD and HC groups showed larger N170 amplitudes in the right hemisphere than in the left hemisphere, while the amplitude showed no hemispheric difference in the apathetic PD group. Holistic facial emotion processing predominantly occurs in the right hemisphere [48]. The undifferentiated N170 amplitude of the bilateral hemisphere and smaller N170 amplitude in the right hemisphere in apathetic PD patients may account for abnormal holistic facial emotion processing. The apathetic patients exhibited delayed N170 latencies for neutral and negative faces in the right temporal site, which is another indication of impairment. As there was no significant difference between the two PD groups for N170 amplitude and latency in the left hemisphere, we speculate that only the right hemisphere plays a particular role in apathy. This idea is supported by a study demonstrating that apathy is more common in patients with right hemisphere damage than left [49]. The negative correlation between the N170 amplitude of the right hemisphere in PD patients and the scores of the SAS in this study may also support this association. Apathy has been associated with neurometabolite changes and glial function in the right temporo-parietal cortex [50]. Meanwhile, metabolic increases in the right fusiform gyrus and hippocampus changed along with improved apathy scores after the use of an apomorphine pump in PD patients [51]. These reports are consistent with the poorer N170 in apathetic patients in the right hemisphere in our study. We also speculate that N170 in the right hemisphere is more sensitive as a reflection of emotion processing for apathetic PD patients. There was no significant difference between the nonapathetic patients and controls for N170 latency, demonstrating the relatively preserved timing for the processing of structurally encoding emotional expressions in nonapathetic patients. The cortical discrimination of emotional faces was associated with executive function [52]. The correlation between the N170 amplitude of the right hemisphere and TMT performance in the apathetic group indicated that executive function may be impaired synchronously with the holistic facial emotion processing. Similar to the behavioural results, there was no significant difference in the latency for positive expressions across the three groups, implying less sensitivity of positive stimuli in reflecting the timing of emotional processing.

## 5. Conclusions

The present findings showed that emotional face processing in apathetic PD patients is impaired in certain aspects based on observations of ERP early time windows. Nonapathetic PD patients, by contrast, showed more selective deficits in abnormal emotional processing. The right hemisphere was predominant in emotional processing and was more sensitive for reflecting impairment in apathetic patients. Furthermore, we would like to investigate other aspects of deficits in social functions to enable targeted early interventions.

## Figures and Tables

**Table 1:** Demographic and neuropsychological data of PD patients and HCs.

	Apathetic group	Nonapathetic group	HC	*p*
Age	64.93 ± 6.47	63.48 ± 6.40	63.76 ± 6.42	0.778
Sex (male), *n* (%)	10 (66.7)	18 (72)	14 (61.9)	0.907
Education (yr)	8.67 ± 3.56	7.80 ± 3.37	8.76 ± 2.98	0.753
Hoehn–Yahr stage	2 (1–3)	2 (1–3)	—	—
UPDRS	18.99 ± 4.22	17.31 ± 5.17	—	—
BDI-II	5.47 ± 2.13	5.44 ± 2.22	4.33 ± 1.68	0.133
SAS	20.33 ± 4.34	7.92 ± 2.31	4.71 ± 1.95	<0.001
Disease duration	6.47 ± 2.26	6.36 ± 1.35	—	—
L-DOPA daily dose (mg)	476.47 ± 78.98	454.80 ± 89.61	—	—
Test scores				
MoCA	28.00 ± 1.31	27.56 ± 1.36	28.33 ± 1.71	0.215
Digit span forward	7.33 ± 1.05	7.36 ± 1.78	8.48 ± 2.11	0.068
Digit spEan backward	3.60 ± 1.18	3.68 ± 1.07	4.33 ± 0.91	0.060
TMT (seconds)				
Part A	76.40 ± 15.71	63.40 ± 12.81	51.71 ± 10.04	<0.001
Part B	188.47 ± 28.05	163.28 ± 33.32	142.00 ± 23.04	<0.001
Word Fluency	15.06 ± 3.56	14.44 ± 3.40	16.38 ± 2.67	0.129

Comparison of demographic data, clinical variables, and neuropsychological tests among the three groups. UPDRS, Unified Parkinson's Disease Rating Scale; BDI-II, Beck Depression Inventory II; SAS, Starkstein Apathy Scale; MoCA, Montreal Cognitive Assessment; TMT, Trail-Making Test.

**Table 2:** Behavioural results in the emotional face categorization task for PD patients and HCs.

Variables	Apathetic group	Nonapathetic group	HC	*χ* ^2^ or *F*	*p*
ACC (%)					
Positive	91.6 (3.9)	91.8 (3.7)	92.2 (2.4)	0.286, 0.867	
Neutral	76.9 (4.5)	78.5 (3.6)	87.7 (2.8)	36.773	<0.001
Negative	72.0 (7.5)	79.3 (9.1)	86.9 (4.8)	23.601	<0.001

RT (ms)					
Positive	698.73 (104.49)	704.20 (106.98)	656.76 (182.53)	0.760	0.472
Neutral	826.67 (112.77)	813.92 (114.24)	704.33 (134.87)	6.161	0.004
Negative	905.93 (149.60)	889.64 (183.75)	728.52 (134.44)	7.568	0.001

The behavioural results of the apathetic PD group, nonapathetic PD group, and HC group are expressed as mean (SD). ACC = accuracy (%); RT = reaction time (ms).

**Table 3:** Amplitudes and latencies of ERP components of PD patients and HCs during emotional face categorization.

Group		Left			Right		
		Positive	Neutral	Negative	Positive	Neutral	Negative
P100							
Amplitude (*μ*V)	Apathetic	4.84 (1.22)	5.48 (1.76)	6.30 (2.06)	5.86 (1.13)	6.91 (1.30)	7.66 (2.40)
	Nonapathetic	4.84 (1.61)	5.19 (1.49)	6.02 (1.50)	7.21 (1.56)	5.87 (1.76)	6.31 (1.53)
	HC	7.34 (1.84)	9.12 (2.01)	7.82 (1.98)	7.31 (2.25)	6.67 (1.40)	6.49 (1.05)
Latency (ms)	Apathetic	142.83 (26.44)	149.83 (25.97)	140.50 (25.23)	141.50 (22.87)	152.33 (28.76)	144.83 (24.63)
	Nonapathetic	122.66 (20.72)	122.83 (17.85)	138.83 (24.29)	127.00 (21.37)	117.67 (21.15)	133.50 (22.81)
	HC	118.60 (16.27)	119.40 (18.18)	121.40 (19.35)	110.00 (14.97)	119.00 (17.10)	116.80 (15.77)

N170							
Amplitude (*μ*V)	Apathetic	12.09 (2.81)	9.97 (2.89)	11.07 (2.85)	12.48 (4.07)	11.39 (3.87)	10.95 (3.43)
	Nonapathetic	12.24 (3.59)	12.76 (3.99)	13.53 (3.58)	15.11 (4.95)	15.83 (5.46)	16.06 (4.81)
	HC	20.07 (6.36)	19.84 (5.68)	21.46 (5.68)	24.38 (7.31)	24.91 (6.44)	25.27 (7.17)
Latency (ms)	Apathetic	200.67 (20.52)	202.50 (32.84)	203.50 (27.38)	206.50 (25.61)	209.83 (25.43)	208.83 (31.17)
	Nonapathetic	183.17 (35.16)	182.67 (35.57)	192.17 (32.71)	187.83 (34.36)	182.50 (31.17)	184.17 (23.80)
	HC	187.20 (43.64)	182.20 (27.04)	189.60 (33.60)	183.00 (32.21)	183.60 (21.63)	183.00 (24.29)

P100 and N170 results from the apathetic PD group, nonapathetic PD group, and HC group are expressed as mean (SD).

## Data Availability

The data used to support the findings of this study are available from the corresponding author upon request.
